# The head shaft angle is associated with hip displacement in children at GMFCS levels III-V - a population based study

**DOI:** 10.1186/s12891-018-2275-4

**Published:** 2018-10-05

**Authors:** L Finlayson, T Czuba, M S Gaston, G Hägglund, J E Robb

**Affiliations:** 1 University of Edinburgh, Edinburgh, Scotland; 2 Epidemiology and Register Center South, Lund, Sweden; 3 Department of Orthopaedic Surgery, Royal Hospital for Sick Children, Edinburgh, Scotland; 4 Lund University Department of Clinical Sciences, Orthopedics, Lund, Sweden; 5 School of Medicine, University of St Andrews, St. Andrews, Scotland

**Keywords:** Children, Cerebral palsy, Hip displacement, Head shaft angle

## Abstract

**Background:**

An increased Head Shaft Angle (HSA) has been reported as a risk factor for hip displacement in children with cerebral palsy (CP) but opinions differ in the literature. The purpose of this study was to re-evaluate the relationship between HSA and hip displacement in a different population of children with CP.

**Methods:**

The Cerebral Palsy Integrated Pathway Scotland surveillance programme includes 95% of all children with CP in Scotland. The pelvic radiographs from 640 children in GMFCS levels III-V were chosen. The most displaced hip was analysed and the radiographs used were those taken at the child’s first registration in the database to avoid the potential effects of surveillance on subsequent hip centration. A logistic regression model was used with hip displacement (migration percentage [MP] ≥40%) as outcome and HSA, GMFCS, age and sex as covariates.

**Results:**

The MP was ≥40% in 118 hips with a mean HSA of 164° (range 121–180°) and < 40% in 522 hips with a mean HSA of 160° (range 111–180°). The logistic regression analysis showed no significant influence of age and sex on MP in this population but a high GMFCS level was strongly associated with hip displacement. An increased HSA was also associated with hip displacement, a 10° difference in HSA for children adjusted for age, sex, and GMFCS gave an odds ratio of 1.26 for hip displacement equal or above 40% (*p* = 0.009) and hips with HSA above 164.5 degrees had an odds ratio of 1.96 compared with hips with HSA below 164.5 degrees (*p* = 0.002).

**Conclusion:**

These findings confirm that HSA is associated with hip displacement in children in GMFCS levels III-V.

## Background

For hip surveillance in cerebral palsy (CP) it is important to identify risk factors for hip displacement both for the treatment of the individual child and to optimise the monitoring programme. Young age and severe limitation of gross motor function as measured with the Gross Motor Function Classification System (GMFCS) are known risk factors.

**Fig. 1:**
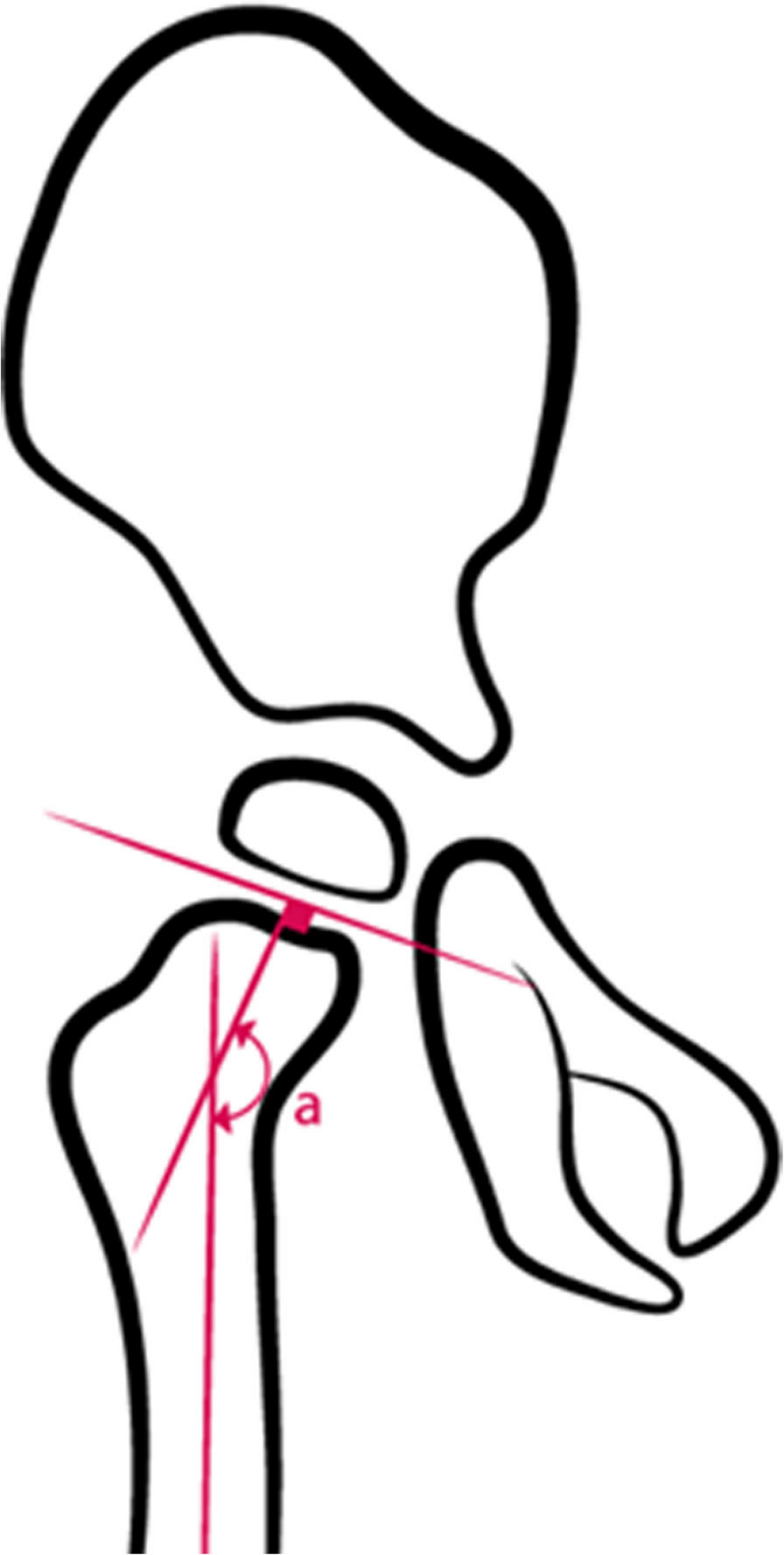
Measurement of the Head Shaft Angle (HSA)

The measurement of the head-shaft angle (HSA, Fig. 1) was popularised by Southwick [1] to measure the degree of slip in slipped upper femoral epiphysis. Foroohar et al. [2] evaluated the relationship between the femoral shaft and head in children with cerebral palsy (CP) and compared the HSA in children with CP with the HSA in typically developing children. They also evaluated the HSA in children with CP who had significant hip displacement. They concluded that the HSA was greater in children with CP and more pronounced in those at risk for displacement and that the evaluation of HSA may be prudent in children with CP.

Lee et al. [3] analysed HSA, Migration Percentage (MP) and the neck-shaft angle (NSA) in 384 patients with CP. They found a higher correlation between NSA and MP than between HSA and MP and concluded that the NSA appeared to be more clinically relevant than the HSA in evaluating proximal femoral deformity in patients with CP. However, the material included children up to 17 years of age some of whom did not have a well demarcated growth plate, making HSA measurement less reliable. Subsequently, van der List et al. [4] reported from a retrospective study that the HSA decreased over time in normal hips and children in GMFCS levels II-III but not in GMFCS levels IV-V, the two groups most at risk of developing hip subluxation. In the same year, van der List et al. [5] concluded from a retrospective cohort that, at age two years, the GMFCS and HSA were valuable predictors for hip displacement, but at the age of four years, only the MP should be used in the prediction of hip displacement. These studies seemed to confirm that the HSA was higher in GMFCS IV-V children at least up to the age of two years, but that the effect of age on the HSA as a predictor of hip displacement was less clear.

Hermanson et al. [6] reported on a total population of children with CP in a defined geographical area followed up for five years or until the development of a MP > 40% of either hip within five years. The use of MP 40% as cut off was chosen as hips with MP > 40 have a high risk for further displacement, indicating the need for surgical intervention [7]. They concluded that a high HSA was a risk factor for hip displacement in children with CP. However, in 2016 Chougule et al. [[8] reported no statistically significant correlation between HSA and hip migration in children with CP aged 3–18 years in GMFCS levels III-V.

These seemingly contradictory reports on the relationship between the HSA, age and GMFCS as a risk factor for hip displacement prompted the present study from another defined population of children with CP. The Cerebral Palsy Integrated Pathway Scotland (CPIPS) surveillance programme includes 95% of all children with CP in Scotland. We have used radiographs from this total population to analyse the effect of HSA on hip displacement defined as MP > 40%.

## Methods

Radiological data from all children in GMFCS levels III-V was used in the analysis. Children receive a pelvic radiograph on first registration in the programme. This is taken in a standardised position which is used across Scotland [9]. Subsequent radiographs are taken according to a protocol based on age and GMFCS. In this study all radiographs taken at first registration into the programme of children in GMFCS levels III-V were used for analysis to avoid potential effects of surveillance on subsequent hip centration. Radiographs showing femoral osteotomy before inclusion in the programme were excluded. Hips were defined as displaced if the MP was ≥40%. The most displaced hip from each child was analysed. The HSA for all radiographs were measured by one observer (LF). The MP for the index radiograph had been previously been recorded in the database by clinicians responsible for the individual care of the children.

Two logistic regression models were used with hip displacement (MP ≥40%) as the outcome. In the first model the following covariates were used: HSA and age (both continuous), GMFCS (categorical, level III as reference), sex (categorical, female as reference). For the second model a ROC curve analysis between HSA and MP ≥40% was done. The ideal cut-off point was selected as the point that maximizes the product of specificity and sensitivity. HSA was dichotomised according to the ideal cutoff point and in the second model the HSA was analysed as a categorical covariate related to this cutoff value. The other covariates were similar to the first model.

The statistical analyses were performed using STATA 13 software.

## Results

**Fig. 2:**
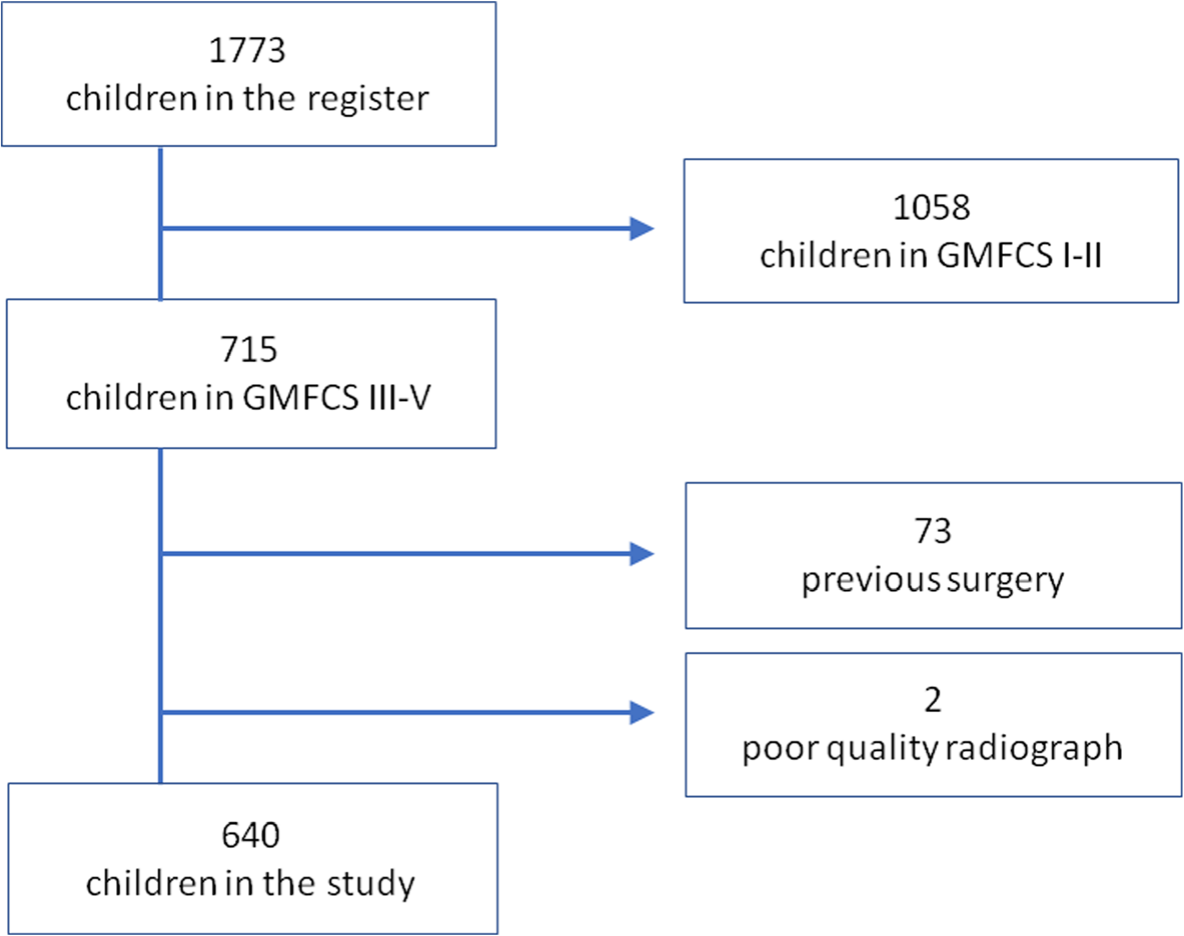
Flow chart describing inclusion and exclusion

There were 640 children with a mean age of 8.2 years (range 0–19 years, 271 female and 369 male) fulfilling the inclusion criteria (Fig. 2). Their distribution by GMFCS level was as follows: III - 160, IV - 184 and V - 296. One hundred and eighteen hips had a MP ≥40% in which the mean HSA was 164° (range 121–180°). Five hundred and twenty two hips with a MP < 40% had a mean HSA of 160° (range 111–180°). The first logistic regression analysis using HSA as a continuous variable showed no influence of age and sex had on MP but a high GMFCS level was strongly associated with hip displacement (Table 1). HSA was also statistically significantly associated with hip displacement. A 10° difference in HSA for patients adjusted for age, sex, and GMFCS gave an odds ratio of 1.26 for hip displacement to a MP > 40% (*p* = 0.009) (Table 1). The ROC curve analysis between HSA and MP ≥40% showed that the ideal cutoff point was HSA =164,5 degrees. The second logistic regression analysis showed that HSA above 164.5 degrees had an odds ratio of 1.96 for MP ≥40% (Table 2).

**Table 1:** Logistic regression estimates on the effect of HSA, GMFCS-level and age on MP using HAS as continuous variable

	Odds Ratio	95% CI		P
HSA^a^	1.26	1.06	1.50	0.009
GMFCS IV	2.86	1.39	5.90	0.004
GMFCS V	4.17	2.13	8.16	0.000
Age^b^	1.03	0.99	1.08	0.174
Sex^c^	0.94	0.62	1.42	0.763

^a^degrees, ^b^years, ^c^female as reference

**Table 2:** Logistic regression estimates on the effect of HSA, GMFCS-level and age on MP with HSA dichotomized

	Odds Ratio	95% CI		P
HSA^a^	1.96	1.29	3.00	0.002
GMFCS IV	2.78	1.35	5.74	0.006
GMFCS V	3.98	2.03	7.80	0.000
Age^b^	1.03	0.99	1.08	0.134
Sex^c^	0.94	0.62	1.42	0.755

^a^HSA < 164,5 degrees as reference ^b^years, ^c^female as reference

## Discussion

This study has confirmed Hermanson et al’s [6] findings that a high HSA is a risk factor for hip displacement in children with CP. Both studies used defined populations of children with CP and thus probably avoided selection biases. The present study does not give prospective information on HSA changes over time, unlike Hermanson et al’s study, because radiological data was taken from radiographs at the time of first registration of children into the surveillance programme. It does, however, provide information on the HSA without the potentially confounding influence of surveillance on hip centration.

The findings of the present study differ from those of Chougule et al. [8] who reported no statistically significant correlation between HSA and hip migration in children with CP aged 3–18 years in GMFCS levels III-V. There are some differences between the two studies which may explain the respective conclusions. Chougule et al. [8] used a linear regression model in their statistical analysis and randomised hips by laterality in the study design. There is a floor and ceiling effect for MP at 0% and 100% that may limit the usefulness of linear regression analysis which is not seen with logistic regression, where a binary outcome of hip ‘displaced’ or ‘not displaced’ was used and set at MP ≥ 40%. Randomisation by laterality may also dilute a study population of hips at risk for displacement. Children in higher GMFCS levels may have pelvic obliquity and the abducted hip would most probably have a MP within normal limits and the opposite, adducted, hip an abnormal MP. For this reason we analysed the most displaced hip in each child. Van der List et al. [5] used both hips in their analyses and also did a logistic regression analysis for displacement with MP ≥40%.

The present study does have limitations. A single observer measured the HSA in all 640 hips. Training in the measurement was given and, reassuringly, Hermanson et al. [10] have reported excellent observer reliability for HSA. The present study is based on the radiographs of children at the time of registration into the CPIPS programme and effects of time or intervention on hip centration or HSA were not considered. The results are only valid for the age range and range of HSA-angle in the material.

## Conclusion

This study, which was based on a total CP population, has confirmed that HSA is associated with hip displacement in children in GMFCS levels III-V.

## Abbreviations

CI: Confidence Interval
CP: Cerebral palsy
GMFCS: Gross Motor Function Classification Scale
HSA: Head Shaft Angle
MP: Migration Percentage
ROC: Receiver operation characteristic

## References

[1] Southwick WO (1967). Osteotomy of the lesser trochanter for slipped capital femoral epiphysis. J Bone Joint Surg Am.

[2] Foroohar A, McCarthy JJ, JJ YD, Clarke S, Brey J (2009). Head-shaft angle measurement in children with cerebral palsy. J Pediatr Orthop.

[3] Lee KM, Kang JY, Chung CY, Kwon DG, Lee SH, Choi IH, Cho TJ, Yoo WJ, Park MS (2010). Clinical relevance of valgus deformity of proximal femur in cerebral palsy. J Pediatr Orthop.

[4] van der List JP, Witbreuk MM, Buizer AI, van der Sluijs JA (2015). The head–shaft angle of the hip in early childhood. A comparison of reference values for children with cerebral palsy and normally developing hips. Bone Joint J.

[5] van der List J. P. J., Witbreuk M. M., Buizer A. I., van der Sluijs J. A. (2015). The prognostic value of the head-shaft angle on hip displacement in children with cerebral palsy. Journal of Children's Orthopaedics.

[6] Hermanson M, Hägglund G, Riad J, Wagner P (2015). Head-shaft angle is a risk factor for hip displacement in children with cerebral palsy. Acta Orthop.

[7] Hägglund G, Lauge-Pedersen H, Persson M (2007). Radiographic threshold values for hip screening in cerebral palsy. J Child Orthop.

[8] Chougule S, Dabis J, Petrie A, Daly K, Gelfer Y (2016). Is head–shaft angle a valuable continuous risk factor for hip migration in cerebral palsy?. J Child Orthop.

[9] Kinch K, Campbell DM, Maclean JG, Read HS, Barker SL, Robb JE, Gaston MS (2015). How critical is patient positioning in radiographic assessment of the hip in cerebral palsy when measuring migration percentage?. J Pediatr Orthop.

[10] Hermanson M, Hägglund G, Riad J, Rodby-Bousquest E (2017). Inter- and intrarater reliability of the head-shaft angle in children with cerebral palsy. J Child Orthop.

